# BCG vaccination and multiple sclerosis risk: A Norwegian cohort study

**DOI:** 10.1177/13524585241230440

**Published:** 2024-02-27

**Authors:** Ola Nakken, Jan Harald Aarseth, Stig Wergeland, Hein Stigum, Haakon E Meyer, Trygve Holmøy

**Affiliations:** Department of Neurology, Akershus University Hospital, Lørenskog, Norway; Norwegian MS Registry and Biobank, Department of Neurology, Haukeland University Hospital, Bergen, Norway/Institute of clinical medicine, University of Bergen, Bergen, Norway; Norwegian MS Registry and Biobank, Department of Neurology, Haukeland University Hospital, Bergen, Norway; Norwegian Institute of Public Health, Oslo, Norway/ Department of Community Medicine and Global Health, University of Oslo, Oslo, Norway; Norwegian Institute of Public Health, Oslo, Norway/ Department of Community Medicine and Global Health, University of Oslo, Oslo, Norway; Department of Neurology, Akershus University Hospital, Lørenskog, Norway/Institute of Clinical Medicine, University of Oslo, Oslo, Norway

**Keywords:** Multiple sclerosis, cohort studies, risk factors, vaccines, Bacillus Calmette–Guérin, epidemiology

## Abstract

**Introduction:**

Bacillus Calmette–Guérin (BCG) vaccination induces long-lasting effects on the adaptive and innate immune systems and prevents development of experimental autoimmune encephalomyelitis and possibly also inflammatory disease activity in multiple sclerosis (MS).

**Objective:**

The objective is to examine if BCG given in early adulthood decreases MS risk.

**Methods:**

From 791,369 (52% females) Norwegians participating in a national tuberculosis screening program from 1963 to 1975, we collected information on BCG vaccination and tuberculosis disease status. Later, MS disease was ascertained through both the Norwegian MS Registry and Biobank and the Norwegian Death Registry. We used logistic regression models to assess the relationship between BCG vaccination and MS risk.

**Results:**

In those BCG vaccinated, mean age at vaccination was 15.6 (standard deviation (SD) = 5.5) years. A total of 2862 (65% females) MS cases were retrieved. Overall, we found no association between MS risk and BCG vaccination. Compared to non-BCG-vaccinated individuals with no signs of tuberculosis infection, odds ratio (OR) for MS was 1.00 (95% confidence interval (CI) = 0.80–1.25) in the BCG-vaccinated group. In those not BCG vaccinated because of latent tuberculosis infection, the corresponding OR was 0.86 (95% CI = 0.66–1.13).

**Conclusion:**

We found no evidence of BCG vaccination or latent tuberculosis infection in young adulthood being linked to MS risk.

## Introduction

A relationship between vaccinations and the risk of developing multiple sclerosis (MS) has not been firmly established.^1,2^ Conclusions from meta-analyses are difficult as the vast majority of studies on this topic have been retrospective case–control studies, which are prone to biases. Prospective and population-based studies focusing particularly on hepatitis B and human papilloma virus have not found an association between vaccinations and MS risk.^3,4^ There was, however, an increased risk of clinical onset of demyelinating disease shortly after any vaccination, suggesting that vaccination may trigger conversion from subclinical to clinically overt disease.^3^

Bacillus Calmette–Guérin (BCG) is an attenuated strain of *Mycobacterium bovis* used extensively to protect against tuberculosis.^5^ Intravesical instillation of BCG is also used as an adjuvant in the treatment of bladder cancer, as BCG infection of epithelial and tumor cells induce both immune activation and antitumor effects.^6^ In addition to heterologous T-cell responses, BCG induces long-lasting metabolic and epigenetic reprogramming of the innate immune system known as trained immunity.^7^ These diverse and persistent effects on immune regulation can also be relevant for treatment and prevention of autoimmune diseases.^8^

A meta-analysis including six human case–control studies did not find a significant change in MS risk after BCG vaccination.^1^ BCG does, however, protect against disease development in animal models including the experimental autoimmune encephalitis model of MS^9^ and has also been studied in prevalent MS. In a baseline controlled trial, one single dose of BCG vaccination reduced the number of new gadolinium-enhancing magnetic resonance imaging (MRI) lesions^10^ and also the proportion of such lesions evolving into persistent T1 hypointense lesions over a 24-month follow-up.^11^ In a placebo-controlled study in patients with a clinically isolated syndrome compatible with the first symptom of demyelinating disease, BCG vaccination significantly decreased the number of new T1-hypointense lesions during the first 6 months.^12^ Those vaccinated also had fewer relapses after 18 months and lower risk of conversion to clinically definite multiple sclerosis (CDMS) over 5 years, suggesting both a rapid and long-lasting effect on disease activity in MS.

We hypothesized that BCG vaccination per se could protect against MS and tested this hypothesis using data from a population-based Norwegian tuberculosis screening program.

## Methods

### Study design and population

As a response to high tuberculosis rates in Norway in the beginning of the 20th century, the Norwegian health authorities initiated large-scale tuberculosis screening and BCG vaccination from 1950 onwards.^13^ All individuals with a negative tuberculin skin test and no signs of tuberculosis disease were strongly encouraged to accept vaccination with BCG. Apart from a small minority of known tuberculosis contacts vaccinated in young childhood, there were generally two arenas for BCG vaccination: (1) vaccination in primary schools at age 12–14 years, and the proportion vaccinated at school in birth cohorts born after 1940 was very high (92%) and (2) vaccination in a tuberculosis screening program. In these repetitive screening rounds, individuals younger than 40 years (50 in some counties) and with no signs of tuberculosis infection were offered BCG vaccination. Vaccination status and year of vaccination, if carried out, was updated after attendance. Citizens who could document a positive tuberculin skin test without prior BCG vaccination were recorded as infected and were neither offered BCG vaccination nor retested. All counties in Norway were included, except the capital of Oslo which had a separate program. Tuberculosis screening attendance was compulsory for every citizen above 14 years of age. Nonattendance was mainly due to “acceptable excuses” such as already under control or treatment for tuberculosis, in military service or in hospital. The overall attendance rate among eligible individuals was 80%–85%, somewhat lower among the youngest and particularly among the oldest persons.^14^ This combined strategy resulted in high BCG coverage among the younger age groups (⩽40 years). Still, some remained unvaccinated due to either confirmed latent tuberculosis infection or refusal to accept vaccination or other unspecified reasons.^15^

BCG was produced at the Bergen State BCG Laboratory (Bergen, Norway) using the Swedish Gothenburg strain until 1973^16^ and thereafter by Statens Serum Institute (Copenhagen, Denmark). Liquid BCG was gradually replaced by freeze-dried BCG between 1959 and 1973.^17^

In this historic population-based cohort study, we studied Norwegian citizens who attended the Norwegian tuberculosis screening programs’ last round between 1963 and 1975, for whom data are computerized. Information from examinations in this last screening round comprise demographic data, results from chest X-rays, the time of the last tuberculin skin test and its result, measured heights and weights, and documentation of BCG vaccination status. From the full screening cohort (*n* = 1,911,598), we first identified participants aged 14–40 years (*n* = 826,057). Individuals younger than 14 years were a selected group screened because of high tuberculosis risk and thereby not included. We excluded individuals older than 40 years because of MS typical debut ages and less accessible MS diagnoses in older birth cohorts. We further excluded participants with BCG vaccination status coded as missing or unknown (*n* = 15,944) and those with suspected lung tuberculosis based on chest X-ray findings (*n* = 18,744). Our final study cohort comprised 791,369 participants.

### Case ascertainment

MS cases were ascertained through both the Norwegian MS Registry and Biobank (from here referred to as “the MS-registry”) and the Norwegian Cause of Death Registry (from here referred to as “the COD Registry”).

The MS-registry is based on informed consent. Its operative period dates back to 2001. The completeness of the registry has gradually increased and is currently 87% of that calculated from an administrative health register (Norwegian Patient Registry).^18^ Cases are registered in the database by local neurologists upon diagnosis (incident cases) or retrospectively based on hospital records and patient recall (prevalent cases). From the MS-registry, we collected information on date of symptom onset and date of diagnosis.

Although most MS cases (83%) retrieved within the MS-registry have back-dated symptom onset to periods prior to the registry’s initiation in 2001, many of our study participants born from 1920 to 1960 died before it was possible to be included in that registry. This may particularly be the case for participants going on to develop severe MS. We therefore also ascertained MS through the COD Registry. We searched all death certificates from 1963 to 2020 containing codes corresponding to MS at any level of cause of death, using the following International Classification of Diseases (ICD) codes; ICD 7 (1963–1968): 345, ICD 8 and 9 (1969–1995): 340, and ICD 10 (1996 onwards): G35. If found in both the MS-registry and the COD Registry, the MS-registry was selected. Out of 2862 MS cases, 1297 (45%) were exclusively found in the COD Registry.

Register linkage was facilitated through the unique personal identification number given to all Norwegian citizens.

### Exposure categories and statistics

We first categorized participants into those BCG vaccinated and not BCG vaccinated. Based on tuberculin skin test information, the latter group was further subcategorized into either latent tuberculosis infection (“spontaneous tuberculin converters” with skin induration size of >3 mm from tuberculin skin test either on previous or current screening round)^19^ or no signs of tuberculosis infection. According to the current recommendations, the latter group should have been BCG vaccinated, but still were not. Also individuals previously BCG vaccinated underwent tuberculin skin tests in this screening program, documenting the immunological response to the vaccine. We treated vaccinated individuals uniformly, disregarding their post-vaccination tuberculin reactivity. Using the non-vaccinated group with no signs of tuberculosis infection as a reference, we compared MS risk to both the BCG-vaccinated group as well as to the group infected with latent tuberculosis (not BCG vaccinated).

A natural way to analyze these data would be to use time to MS-diagnosis as the outcome in a survival model. But nearly half of the MS cases were ascertained through the COD Registry and therefore had missing time of diagnosis. Instead, we used logistic regression to calculate odds ratios (ORs) and their 95% confidence intervals (CIs). Participants entered the study at the last screening round and were followed up until November 2020. The youngest persons (14 years) screened at the last year of the inclusion period (1975) must be at least 59 years old at end of follow-up. The risk of MS onset at age over 59 years is very low. Furthermore, the proportion of cases is small (0.36%). We therefore expect ORs from a logistic regression to be similar to hazard ratios from a survival model. We first calculated ORs adjusted for sex and birth cohort (continuous) which we considered being the most important common determinants for both MS risk and BCG status. Age at screening (continuous) and county (categorical) was included as covariates in our fully adjusted model. Between sexes heterogeneity was tested using a likelihood ratio test between the multivariable adjusted model and a model including an interaction term of exposure categories and sex.

We performed a sensitivity analysis in order to minimize the risk of including MS cases with onset prior to BCG vaccination. Here, we restricted the vaccinated group to those vaccinated before 18 years of age.

In the logistic regression model above, the time to MS-diagnosis is not used. To see if the ORs from the logistic model would differ from hazard ratios from a survival model using the time to MS symptom onset, we calculated hazard ratios (HRs) and their CIs from a supplementary analysis using interval-censored survival model (stintreg-command in Stata). Each non-case contributed follow-up time from the date of tuberculosis screening (1963–1975) to the date death, emigration, or the end of study follow-up (November 2020), whichever came first. Follow-up time for cases retrieved from the MS-registry was calculated from the date of tuberculosis screening to the date of symptom onset. Cases from the COD Registry were handled with interval censoring where lower possible follow-up was set to time of tuberculosis screening and upper possible follow-up was set to time of death. An exponential distribution was used to generate follow-up times for these patients. Model covariates were then specified as described for the logistic analyses.

## Results

A total of 791,369 (52 % females) screening participants met our eligibility criteria. Mean age at tuberculosis screening was 26.2 (standard deviation (SD) = 7.9) years. The gross majority was BCG vaccinated (89%), where mean age at vaccination was 15.6 (SD = 5.5) years. Those vaccinated were generally younger at screening and born in more recent birth cohorts compared to those not vaccinated (Table 1). These differences were, however, less clear in the subgroup of non-vaccinated individuals who remained uninfected. This group comprised more females than the other two exposure categories (Table 1). A total of 2862 (65% females) MS cases were retrieved in either the MS-registry or the COD Registry.

**Table 1.** Baseline characteristics by BCG vaccination and *Mycobacterium tuberculosis* infection status.

	BCG vaccinated	Non-BCG vaccinated, not infected^a^	Non-BCG vaccinated, infected^a^
Participants	707,154	21,525	62,690
Females (%)	366,431 (52)	15,196 (71)	31,639 (50)
Birth year, mean (SD)	1943 (8.0)	1940 (9.6)	1937 (8.2)
Age at screening, mean years (SD)	25.6 (7.7)	27.9 (8.9)	31.6 (7.5)
Age at vaccination, mean years (SD)	15.6 (5.5)	n/a	n/a
Body mass index, mean kg/m^2^	22.7	23.1	23.7

BCG: Bacillus Calmette–Guérin; SD: standard deviation; n/a: not applicable.

Norwegian citizens aged 14–40 years who attended the last round of a nationwide tuberculosis screening program (1963–1975). MS cases identified through both the Norwegian MS Registry and Biobank and the Norwegian Cause of Death Registry.

a *M. tuberculosis* infected.

Overall, we found no association between MS risk and BCG vaccination. Compared to non-BCG vaccinated individuals with no signs of tuberculosis infection, OR was 1.00 (95% CI = 0.80–1.25) in the BCG-vaccinated group. The corresponding OR for those infected with latent tuberculosis (not BCG vaccinated) was 0.86 (95% CI = 0.66–1.13) (Table 2). There was no effect difference between genders (*p* = 0.25). We also performed analyses stratified by birth cohorts. Compared to younger birth cohorts (1940–1959), older birth cohorts (1920–1939) were characterized by (1) lower proportion of vaccinated individuals (82%), (2) fewer MS cases identified with symptom onset within the MS-registry (79% found exclusively in the COD Registry), and (3) older age at vaccination (mean = 19.7 (SD = 6.7) years). In these older birth cohorts, there were rather few cases in our reference category comprising non-BCG-vaccinated, uninfected individuals. Still, ORs for those BCG vaccinated were similar in old and young birth cohorts (Table 2). The ORs for the infected, non-BCG-vaccinated group were slightly different between birth cohort categories, deviating more from one in older birth cohorts. Still, no clear association with MS risk was seen in any birth cohort category.

**Table 2.** Risk (OR) of MS according to BCG vaccination, *Mycobacterium tuberculosis* infection status, and birth cohort.

Exposure category	Participants	Cases	Sex and birth year-adjusted OR (95% CI)	Multivariable adjusted^a^ OR (95% CI)
All				
Non-BCG vaccinated, not infected^b^	21,525	82	Reference	Reference
BCG vaccinated	707,154	2616	0.98 (0.79–1.22)	1.00 (0.80–1.25)
Non-BCG vaccinated, infected	62,690	164	0.82 (0.63–1.07)	0.86 (0.66–1.13)
Born 1920–1939				
Non-BCG vaccinated, not infected	10,542	32	Reference	Reference
BCG vaccinated	238,424	667	0.99 (0.69–1.41)	1.03 (0.72–1.48)
Non-BCG vaccinated, infected	43,281	92	0.78 (0.52–1.16)	0.84 (0.56–1.26)
Born 1940–1959				
Non-BCG vaccinated, not infected	10,981	50	Reference	Reference
BCG vaccinated	464,722	1949	0.97 (0.73–1.28)	0.98 (0.74–1.30)
Non-BCG vaccinated, infected	19,408	72	0.88 (0.61–1.27)	0.92 (0.64–1.32)

OR: odds ratio; CI: confidence interval; BCG: Bacillus Calmette–Guérin.

Norwegian citizens aged 14–40 years who attended the last round of a nationwide tuberculosis screening program (1963–1975). MS cases identified through both the Norwegian MS Registry and Biobank and the Norwegian Cause of Death Registry.

a Adjusted by age, birth year, county, and sex.

b *M. tuberculosis* infected.

We performed a sensitivity analysis where risk of including MS cases with disease onset prior to BCG vaccination was minimized. We restricted the vaccinated group to those with age at vaccination less than 18 years. Here, mean age at vaccination was 13.1 (SD = 2.3) years. The results did not change substantially (Table 3).

**Table 3.** Risk (OR) of MS according to BCG vaccination and *Mycobacterium tuberculosis* infection status.

Exposure category	Participants	Cases	Sex and birth year-adjusted OR (95% CI)	Multivariable adjusted^a^ OR (95% CI)
Non-BCG vaccinated, not infected^b^	21,525	82	Reference	Reference
BCG vaccinated	415,016	1612	0.97 (0.78–1.22)	0.99 (0.79–1.24)
Non-BCG vaccinated, infected	62,690	164	0.82 (0.63–1.07)	0.86 (0.66–1.12)

OR: odds ratio; CI: confidence interval; BCG: Bacillus Calmette–Guérin.

Norwegian citizens aged 15–40 years who attended the last round of a nationwide tuberculosis screening program (1963–1975). BCG vaccinated restricted to those vaccinated before age 18 years. MS cases identified through both the Norwegian MS Registry and Biobank and the Norwegian Cause of Death Registry.

a Adjusted by age, birth year, county, and sex.

b *M. tuberculosis* infected.

In our supplementary analysis using a survival model with interval censoring for those cases ascertained within the COD registry, results were similar to those generated from the logistic model. Thus, compared to non-BCG-vaccinated individuals with no signs of tuberculosis infection, hazard ratio (HR) for MS was 0.96 (95% CI = 0.77–1.21) in the BCG-vaccinated group and 0.86 (95% CI = 0.66–1.13) in those infected with latent tuberculosis.

## Discussion

In this register-based cohort study using data from a nationwide tuberculosis screening program, neither those BCG vaccinated nor those infected naturally with *Mycobacterium tuberculosis* differed from the non-vaccinated and uninfected population in terms of MS risk.

Our findings are in accordance with previous case–control studies.^20–25^ These studies have been relatively small hospital-based and retrospective case–control studies, comprising altogether 536 MS cases and 751 controls from the general population. One large Canadian cohort study has been conducted.^26^ Different from our study population, vaccinated Canadians were almost always vaccinated during infancy, and MS cases were ascertained exclusively through administrative health data. They found that BCG vaccination was associated with increased MS risk for later onset overall MS, but not for MS cases with ⩾1 disease-modifying treatment claim, in their study defined as relapsing remitting multiple sclerosis (RRMS).^26^ Although information on MS subtypes exists within the Norwegian MS Registry, we did not distinguish between RRMS and overall MS. First, restricting analysis to cases known to have RRMS (MS-registry only) would dramatically impact the power of the analysis (RRMS comprised 72% of incident cases, and 37% of all MS cases). More importantly, separating RRMS from primary progressive multiple sclerosis (PPMS) or overall MS is probably not decisive from an etiological viewpoint.^27^ The positive association between BCG and late onset overall MS risk found in the Canadian study was not seen in our population. This could be explained by differences in ages at vaccination or case ascertainment.

The tuberculosis skin test after BCG vaccination represents a model to study the strength of a response to a recall antigen. In a previous study using data from the same cohort but restricted to those BCG-vaccinated only, a strong tuberculosis skin test after BCG vaccination was associated with reduced risk of developing MS.^28^ These results suggest that skewed T-cell-mediated immunity precedes MS onset by many decades. The previous study did, however, not include unvaccinated individuals, and it did not address whether BCG vaccination per se effect MS risk. The results were therefore not at odds with those of this study.

The possibility of vaccines triggering MS have long been debated, yet largely refuted with increasing number of negative studies.^29^ The inverse concept of a potentially protective effect from vaccines on risk of MS and other autoimmune diseases is more recent. BCG may exert a non-specific protective effect from infections through a mechanism independent of adaptive immunity. Trained immunity is a long-term functional reprogramming of innate immune cells following an encounter with a primary stimulus, which results in an augmented response against a secondary challenge.^30^ Any particular modulation of host responses to Epstein–Barr virus, a known risk factor for MS,^31^ is not known.^32^

Our study has limitations. Ascertainment of MS in the Norwegian MS Registry is biased toward more recent birth cohorts. In our study population, the most recent birth cohorts were also more likely to be BCG vaccinated. In addition to incomplete case finding of older birth cohorts within the MS-registry, the time lag between the tuberculosis screening in the 60s and 70s and the possible inclusion in the MS-registry from 2000 onwards could result in a survival bias, where only more benign MS forms were ascertained. To minimize this possibility, we therefore included MS mortality in the COD registry for case finding of more severe MS forms. Still, the sensitivity of the COD registry for ascertaining MS is probably also better for more recent birth cohorts.^33^ As infected individuals were largely over-represented in older cohorts, we suspect that the tendency of a weak decrease in MS risk observed among the infected individuals indeed represent ascertainment bias rather than any immunological protective effect. Near half of cases were ascertained from the COD Registry and did not have information on symptom onset, hence they could possibly have been vaccinated after symptom onset. However, in sensitivity analysis restricting the vaccinated population to those receiving vaccine before age 18 years, when MS onset is rare,^34^ results remained similar. Also, a supplementary analysis using a survival model with interval censoring for those cases ascertained within the COD registry yielded similar results to those generated from our main analysis not including time to MS symptom onset.

The non-vaccinated and non-infected group comprised rather few cases. A possible weak decreased risk for MS in BCG vaccinated compared to those non-vaccinated can therefore not be excluded. Ultimately, as for all observational studies, we cannot rule out bias from unmeasured confounders. Non-vaccinated individuals may represent a selected group. If this potential selection relates to factors also modifying MS risk, a true association may have been diluted. A previous study on the duration of BCG protection against tuberculosis in the same screening cohort^35^ reported that the distribution of socioeconomic indicators available, including educational attainment, occupation, as well as local tuberculosis rates did not vary substantially between BCG vaccinated and non-infected, non-vaccinated individuals.

The major strengths of our study is the population-based design, the size of the population, objective information on BCG vaccination gathered prior to disease onset and long follow-up. Comparing the case proportion (0.36%) in our study population to lifetime MS risk^36^ suggests adequate case finding through two independent sources.

In conclusion, we found no evidence of BCG vaccination or latent tuberculosis infection in young adulthood being linked to MS risk.
